# Improved Walking Through an Aperture in a Virtual Environment Transfers to a Real Environment: Introduction of Enriched Feedback and Gradual Increase in Task Difficulty

**DOI:** 10.3389/fspor.2022.844436

**Published:** 2022-03-04

**Authors:** Yuki Suda, Kazunobu Fukuhara, Kazuyuki Sato, Takahiro Higuchi

**Affiliations:** Department of Health Promotion Science, Tokyo Metropolitan University, Tokyo, Japan

**Keywords:** virtual reality, obstacle avoidance, stepping in place, motor learning, older adults

## Abstract

Virtual reality (VR) could be used to set up a training protocol to improve one's collision-avoidance behavior. In our previous study, we developed a VR system for training older individuals to walk through an aperture in a manner that is both safe (i.e., no collision) and efficient (i.e., no exaggerated behavior to ensure collision avoidance). In the present study, we made several modifications to the VR system in terms of enriched feedback (vibratory stimulation for virtual collisions and the addition of positive feedback for successful trials) and gradual increase in task difficulty during training to strengthen the skill transfer. Nineteen older adults (74.4 ± 5.3 years of age) and 21 younger adults (25.1 ± 5.0 years of age) participated. They were randomly assigned to one of two training groups: the intervention group (older: *n* = 10; younger: *n* = 10) or the control group (older: *n* = 11; younger: *n* = 9). The experiment consisted of pre- and post-training tests in a real environment and training in a VR environment. During training, participants held a horizontal bar while stepping in place as if a VR image on the screen were moving in response to their stepping. Participants in the intervention group tried to pass a narrow aperture without collision while attempting to minimize their body rotation to avoid collision as much as possible. The criterion upon which the collision-avoidance behavior was regarded as successful became incrementally more demanding as participants successfully met the previous criterion. Participants in the control group passed through a very wide aperture, so that collision-avoidance behavior was unnecessary. A comparison between pre- and post-training test performances showed that, for both older and younger adults in the intervention group, the spatial margins became significantly smaller, while the success rate remained unchanged. For those in the control group, neither the spatial margin nor the success rate was improved. These results suggest that the three modifications made for the VR system contributed to improvement of the system and helped participants transfer the behavior learned from the VR environment to real walking.

## Introduction

In recent years, virtual reality (VR) has been widely used to reduce fall risk and provide locomotor training for older adults (Mirelman et al., 2011, 2016; Maillot et al., 2017; LoJacono et al., 2018; Cavallo et al., 2019; Kim et al., 2019; Kondo et al., 2021). VR-based training, which simulates a real environment, can improve both motor and cognitive function, which are important factors in preventing falls (Jaffe et al., 2004; Mirelman et al., 2016; Kimura and van Deursen, 2020). For instance, Mirelman et al. (2016) showed that for older adults, treadmill training augmented by virtual reality improved not only locomotor ability but also cognitive ability. These results suggest that VR is a potential tool to provide locomotor training for older adults.

VR is particularly useful in setting up a training protocol to improve one's collision-avoidance behavior. In a VR environment, there is no physical contact with obstacles, so participants are free from the issues induced by collisions, such as injury, tripping, and falling (Maillot et al., 2017; Cavallo et al., 2019). The fact that there is no physical contact with obstacles in a VR environment is also advantageous for experimentally manipulating feedback on the results during training (Jaffe et al., 2004; Kim et al., 2019). Contact with an obstacle is powerful feedback to inform the participant that the behavior to avoid collision was insufficient (negative feedback). Therefore, ensuring no physical contact during training helps experimenters avoid producing individual differences in the effect of the training due to differences in the frequency of contact.

Recently, Kondo et al. (2021) developed a VR system to train older individuals to walk through an aperture in a manner that is both safe (i.e., no collision) and efficient (i.e., no exaggerated behavior to ensure collision avoidance). Notably, Kondo et al. aimed at training older adults to behave not only safely but also efficiently because they believed that repeatedly taking a cautious strategy prevents older adults from fine-tuning their behavior in response to environmental changes, which will eventually lead to reduced ability in adaptive locomotor adjustment. In their system, the VR image of a walking path and an aperture was presented to participants by projecting it onto a large screen using a three-dimensional (3D) stereo projector. With this system, participants were able to see their own body through peripheral vision. Because participants could learn the spatial relationship between their own bodies and the environment during training, what they learned in the VR environment was expected to be transferred to their behavior in a real environment, where accurately perceiving the spatial relationship between their own bodies and the environment is necessary for safe and efficient behavior. The results showed that training older individuals in their VR system led participants to modify their behavior to move efficiently during real walking. However, at least some participants experienced frequent collisions regardless. These results suggest the necessity of improving the VR system to lead older participants to behave both safely and efficiently.

The aim of the present study was to revisit the study of Kondo et al. (2021), with some modifications to enrich feedback and to gradually increase task demand, to lead older and younger adults to walk through an aperture both safely and efficiently in a real environment. We have made three modifications in the VR system. First, when a virtual collision occurred, a vibratory stimulation on the arm was newly introduced to increase the perceived reality of the collision. In Kondo et al., the verbal information (“Left hit” or “Right hit”) was shown on the screen. Based on recent reports showing that motor learning using VR was not effective under a non-realistic VR environment (Grassini et al., 2020, 2021), we introduced a new vibratory stimulation to enhance the reality of the collision.

Second, new feedback was provided when participants successfully met the criterion of walking through an aperture in a manner that is both safe and efficient. In Kondo et al., feedback was only presented upon collision; therefore, participants were not sure whether their behavior was efficient. Kim et al. (2019) reported the successful transfer of training in the VR environment to locomotor behavior in a real environment, in which feedback was provided not only at the time of collision when stepping over an obstacle, but also at the time of success (at the minimum margin between the toe and the obstacle) to reinforce their behavior.

Finally, we incrementally reduced the critical value of the spatial margin within which the collision-avoidance behavior was regarded as successful in order to gradually increase the task difficulty (termed “gradually demanding training”). Some studies have reported more efficient skill retention and transfer for gradually demanding training than for general training during which the task difficulty remained unchanged (Malfait and Ostry, 2004; Kluzik et al., 2008; Torres-Oviedo and Bastian, 2012). Moreover, the gradually demanding training could reduce cognitive demand (Sawers and Hahn, 2013; Sawers et al., 2013) and lead participants to easily reinforce their behavior. We expect to provide more effective training by adjusting the difficulty in meeting the criterion step by step.

In the present study, we newly tested younger adults, as well as older participants. A few previous studies has shown that the effectiveness of training for the improvement of locomotor behavior was dependent on the age of participants, showing lesser improvement throughout training in older adults (Van Ooteghem et al., 2009; Maillot et al., 2017). Based on these studies, one might assume that the VR training used by Kondo et al. (2021) was not fully effective when older adults were tested. To explore this issue, we tested not only older adults but also younger adults to see if there was any difference between the two groups in the effectiveness of the current VR training. As in the study by Kondo et al. (2021), the effectiveness of the VR training was evaluated to see if the collision-avoidance behavior was improved through training in a manner that is both safe and efficient. That is, the effectiveness was quantified in terms of the collision rate (the lower, the more effective) and spatial margin created between the edge of the hand-held bar and the edge of an aperture (the smaller, the more effective).

## Materials and Methods

### Participants

Twenty-one older adults (9 males and 12 females, age = 74.4 ± 5.3 years) and 19 younger adults (16 males and 3 females, age = 25.1 ± 5.0 years) participated. Each older and younger adult was randomly assigned to one of two training groups: the intervention group (older adults: *n* = 10; younger adults: *n* = 10) or the control group (older adults: *n* = 11; younger adults: *n* = 9). We checked on a self-reported basis that all participants had normal or corrected-to-normal vision, no current musculoskeletal injuries, and no neurological disorders. Testing was approved by the Ethics Committee of Tokyo Metropolitan University, Japan (H31–98). Written informed consent was obtained from all participants in accordance with the Ethics Committee of Tokyo Metropolitan University and the Declaration of Helsinki.

### Tasks, Apparatus, and Procedures

**Figure 1:**
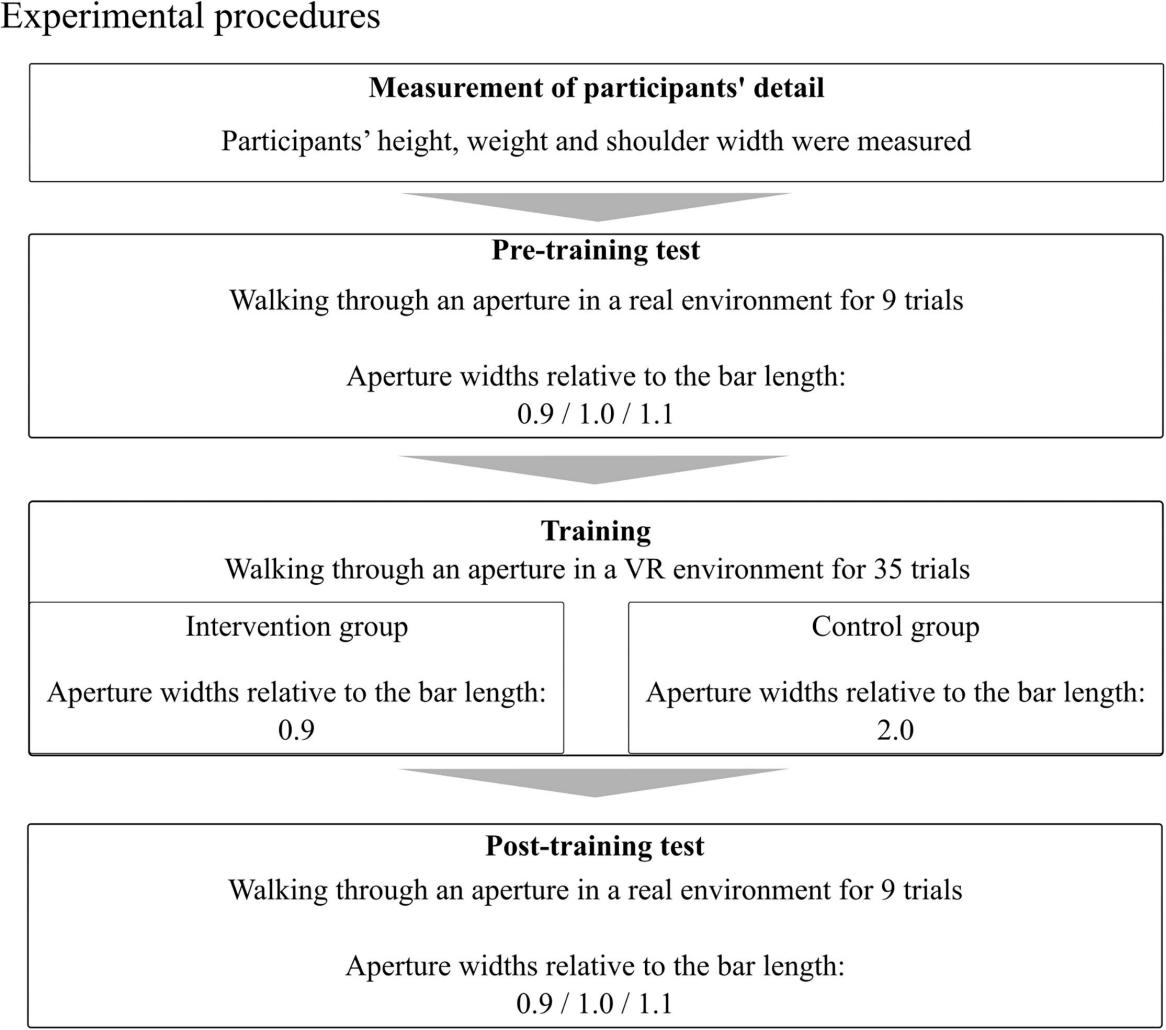
Experimental procedures of this study.

The tasks, apparatus, and procedures were the same as those used by Kondo et al. (2021), with some modifications. The experiment consisted of four parts (Figure 1): measurement of participants' details, a pre-training test in a real environment, training in a VR environment, and a post-training test in a real environment.

### Measurement of Participants' Details

Participants' height and shoulder width were measured in cm, and participants' weight was measured in kg. For older participants, cognitive function was assessed using the Mini–Mental State Examination (MMSE) (Folstein et al., 1975), and the mobility function was assessed using the Timed Up and Go (TUG) test (Podsiadlo and Richardson, 1991).

### Pre- and Post-training Tests in a Real Environment

**Figure 2:**
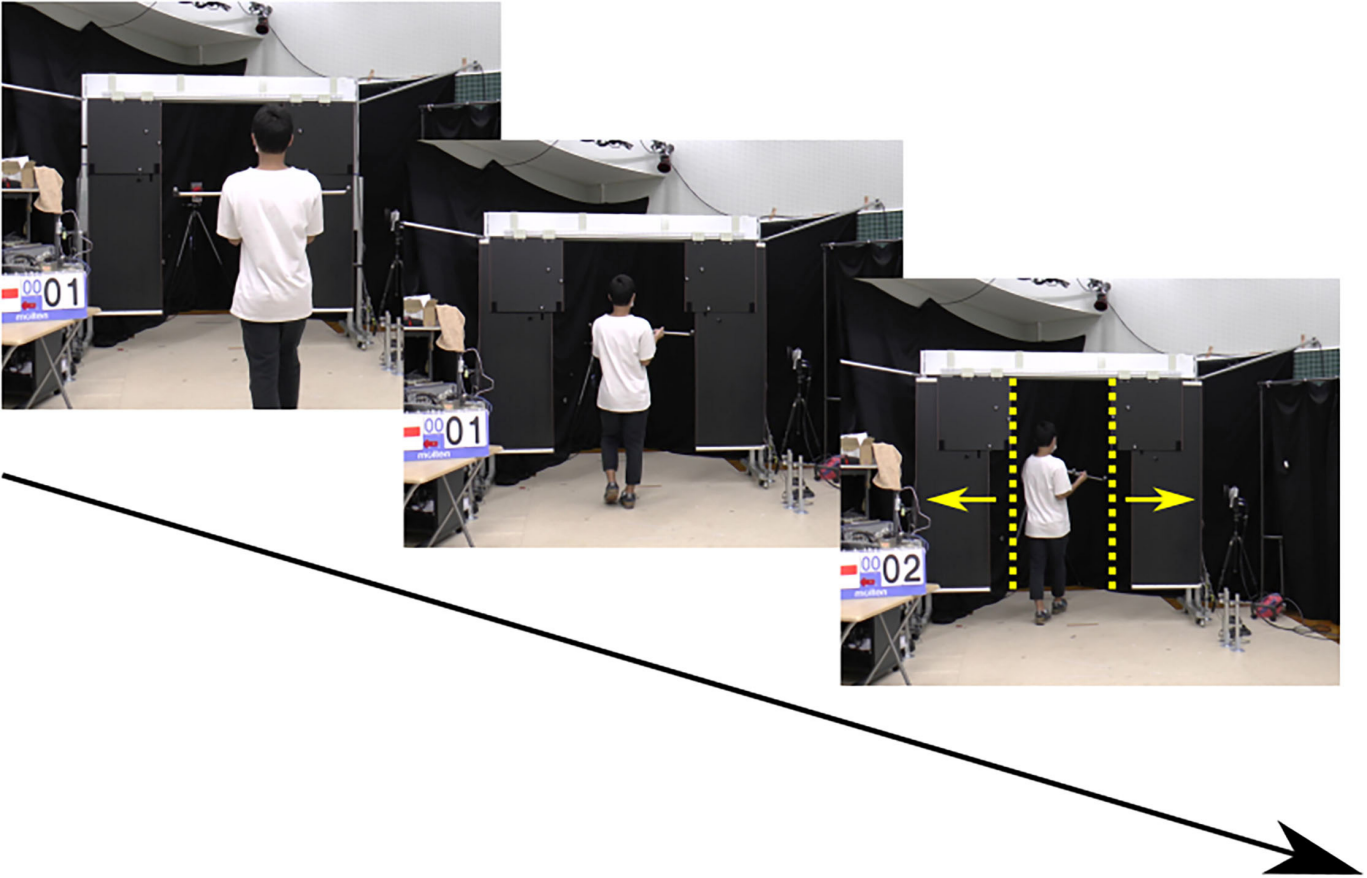
Real environment for pre- and post-training tests.

**Figure 3:**
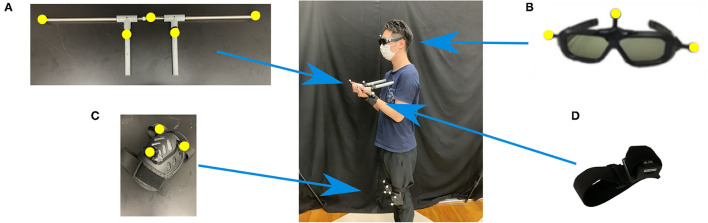
Marker setup and equipment: **(A)** horizontal bar, **(B)** stereo shutter glasses, **(C)** knee pad, and **(D)** vibration stimulator. Yellow markers show reflexive markers.

We conducted tests of pre- and post-training performance along a straight 5.5 m path in a real environment (Figure 2). A custom-made, moving doorway apparatus (Uchidadenshi Co., Japan) was made of two long boards (0.6 m wide × 1.75 m tall) and two short boards (0.6 m wide × 0.6 m tall) and was located 4 m from the starting position. The optical switch, which was used to widen the doorway before participants passed through the aperture to avoid collision, was positioned 0.7 m in front of the aperture. To prevent any feedback to participants regarding their success or failure, the aperture was made wider just before participants passed through it (0.7 m from it) so that actual collisions did not occur. Participants held a horizontal bar while walking. The horizontal bar consisted of a long rod (91 cm wide, 1.6 cm in diameter) and two extra rods (23 cm long, 2.3 cm in diameter) that were attached perpendicularly to the long rod (18 cm apart from each other) with T-shaped connectors (Figure 3A). To maintain the horizontal alignment of the bar and the body in the front dimension while walking, participants gripped each T-shaped connector with one hand and anchored the edges of the rods to the body at the chest. A three-dimensional motion analysis system (OQUS 300, Qualisys, Sweden) with 13 cameras was used to analyze kinematic data relating to the behavior of walking through an aperture. Five reflective markers on the horizontal bar and six reflective markers on the moving door were tracked.

Participants tried to pass through the aperture without collision and were allowed to rotate their shoulders/trunk, but only by as little as possible at the time of crossing (Figure 2). Three different aperture widths were randomly presented: 0.9, 1.0, and 1.1 times the bar width held by the participants. Participants performed a total of nine main trials (three trials for each of three aperture widths). Prior to performing the main trials, participants performed three practice trials (one trial for each of the three aperture widths) to familiarize themselves with the task.

### Training in a VR Environment

Computer graphics of a walking path and an aperture were created with VR-authoring software (Omega Space, Solidray Co., Ltd., Japan). These images were projected using a 3D stereo projector (Sight 3D, Solidray Co., Ltd., Japan) onto a 3.25 m wide × 2.44 m tall screen. Participants wore stereo shutter glasses (3D vision, NVIDIA Corp., USA) with three reflexive markers (Figure 3B) to view the stereoscopic images. Participants also wore knee pads with three reflexive markers for each to track their stepping behavior (Figure 3C). The same horizontal bar (91 cm in length) was used in the VR environment as in the real environment. The reflexive markers were tracked using a 3D motion analysis system (OptiTrack Flex13, NaturalPoint, USA) with five cameras. The 3D motion analysis software (Motive, OptiTrack Japan, Ltd., Japan) was used to reflect participant's movements obtained from the 3D motion analysis system in a VR environment with a time delay of about 65 ± 16 ms. A custom-made vibration stimulator (Solidray Co., Ltd., Japan; Figure 3D) was placed on either the right or left wrist to provide vibratory stimulation (the frequency was 116 Hz) as tactile feedback about virtual collisions.

**Figure 4:**
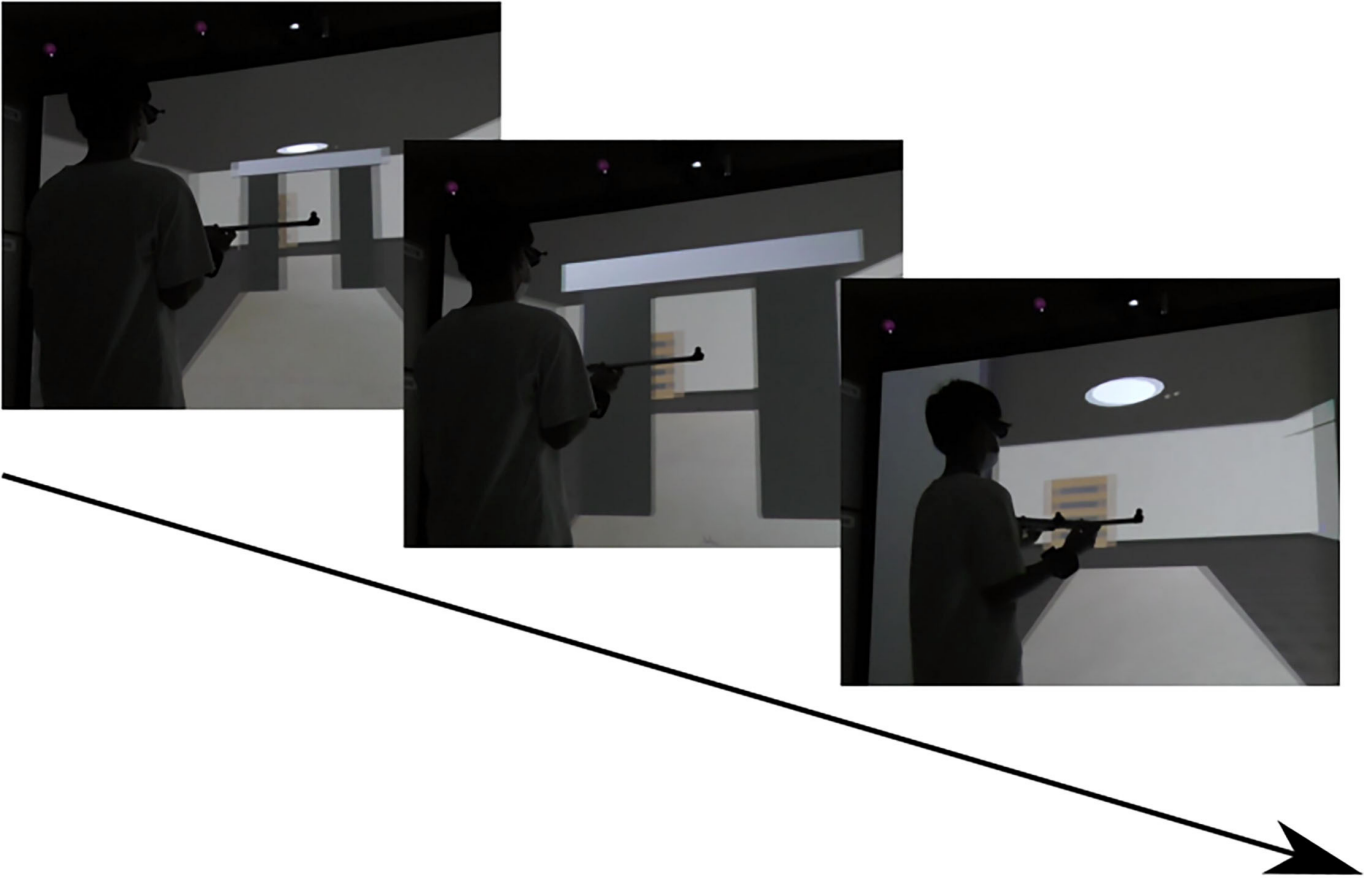
VR environment for training.

The procedures for the VR training of the intervention groups were as follows. In the intervention groups, participants were asked to stand 1.5 m in front of the large screen and start stepping in place as if the VR image on the screen were moving in response to their stepping (Figure 4). The VR image was presented to them with a perception of forward self-motion at 1.00 m/s, which was slower than in Kondo et al. (2021) (1.12 m/sec). During VR training, participants held the long horizontal bar. Participants were asked to pass through the aperture without collision and to minimize the spatial margin created between the bar and the inner edge of the board. The aperture width was 0.9 times the length of the bar held by participants. Participants performed a total of 35 main trials for the aperture width. When a virtual collision between the horizontal bar and either the right or left side of the inner edge of the board occurred, both visual and tactile feedback was presented to the participants. The visual feedback was the phrase “Left HIT” or “Right HIT” presented at the top left or top right of the screen, respectively. The tactile feedback, which was newly introduced in the present study, was a 0.2-s vibration applied to either the right or left wrist. Vibration stimulators were placed around the wrist rather than the arm to mimic the tactile information produced by the contact between the hand-held bar and the edge of the aperture.

The word “Good!!!” was also displayed on the screen as positive visual feedback when the participants successfully met a criterion. The criterion was to walk through an aperture while the spatial margin created between the inner edge of the board and the hand-held bar was within the critical value for each trial. The spatial margin was measured on both sides of the doorway; positive feedback was given to the participants when the spatial margin calculated on both sides met the criterion. The critical value was initially set to 20 cm and then changed gradually according to the following rules: (a) When the participants successfully met the criterion, the critical value was reduced by 2 cm (e.g., when they successfully met the first criterion, the critical value was changed to 18 cm). The critical value was reduced by 2 cm each time participants met the criterion. Theoretically, the criterion value became smaller until reaching zero if participants continuously meet the criterion. (b) When they were unsuccessful, the critical value remained unchanged. (c) When they failed to meet the criterion for two consecutive trials, the critical value was increased by 2 cm, but the value then remained unchanged even if the participants further failed to meet the criterion. The latter rule was necessary to prevent meeting the critical value from being too easy as a result of the participants' trial and error.

The procedures for participants in the control group were basically the same as for those in the intervention group, except that the width of the aperture was always 2.0 times the bar width, so that body rotation was unnecessary to avoid collision, and no feedback was presented. Prior to the main trials, participants performed nine practice trials to familiarize themselves with the task (three trials: the width of the aperture was 2.0 times the bar width; six trials: the width of the aperture was 0.9 times the bar width).

### Data Analyses

A Mann–Whitney test was used to compare participants' characteristics (age, height, weight, shoulder width, and MMSE and TUG scores), excluding gender. A Pearson's chi-squared test was conducted to compare the gender ratio.

To determine whether collision-avoidance strategies were improved in terms of both safety and efficiency after the VR training, two main dependent variables, the collision rate and the spatial margin, were collected during the pre- and post-training test phase. The collision rate during each phase was defined as virtual collision between the inner edge of the board and the horizontal bar because the aperture opened wider before the participants passed through it, and physical contact with the board did not occur (Kondo et al., 2021). The spatial margin, which was defined as the distance between one inner edge of the board and the edge of the horizontal bar on the same side, was calculated at the time of aperture crossing on one side of the horizontal bar (Higuchi et al., 2012). The spatial margin was obtained only from successful trials (i.e., trials with no collisions).

To investigate more deeply the behavior of walking through an aperture, two additional dependent variables, the mean absolute angle of body rotation and the relative distance between mid-body and mid-doorway at aperture crossing, were also collected only from successful trials during the pre- and post-training test phase. The absolute angle of body rotation at the time of aperture crossing was defined as the absolute angle between the horizontal bar and the door (Kondo et al., 2021). To determine whether the mid-body position became closer to the inner edge of the board at the time of doorway crossing, the relative distance between the midsagittal position of the body and the center of the aperture at the time of doorway crossing was obtained for each pre- and post-training test phase (Higuchi et al., 2006). Positive values indicated that participants passed closer to the inner edge of the board than to the center of the aperture, and negative values indicated that participants passed farther from the center of the aperture. The smaller the value, the farther from the inner edge of the board their passing position.

The main dependent variables (collision rate, spatial margin, absolute angle of body rotation, and relative distance between mid-body and mid-doorway) were statistically tested using a three-way (age × training group × phase) analysis of variance (ANOVA) with repeated measures by phase (pre- and post-training tests). Regarding the collision rate, the distribution was not normal, which negated the assumption of using the ANOVA. To deal with the issue, we adjusted the collision rate data by using the arcsine transformation for the analysis.

The main hypothesis of the study—that VR training could help participants to transfer the behavior learned in the VR environment to real walking—was related to the two-way interaction between the effect of the training group and the phase interaction. For this reason, *post-hoc* comparisons were performed using the Bonferroni method only when significant interaction was found for the training group × phase interactions (significance level was corrected to 0.0125). The significance threshold was set to 0.05. The software package SPSS (version 27.0) was used to conduct all statistical analyses.

To discuss the quality of the VR training for participants in the intervention group, their performance during the training session was evaluated using two measurements. First, to see how well participants met the critical value throughout the training trials, the mean value of the critical value was sampled. Second, the positive feedback signals (i.e., the trials in which the word “Good!!!” was given as feedback) throughout 35 training trials were counted. To verify whether improvement was observed in the former or latter half of the trials, the positive feedback signals in the first half (trials 1–17) and in the second half (trials 18–35) were also counted. Third, the behavioral characteristics of the participants during unsuccessful trials (i.e., the trials in which collision occurred) were described in terms of the absolute body rotation angles and the relative distance between mid-body and mid-doorway at aperture crossing. No statistical analyses were made regarding these four measurements.

## Results

### Participants' Characteristics

The participants' characteristics are summarized in Table 1. For both older and younger adults, no significant differences between the training groups were found in any of the measurements. The MMSE scores of all older adults were above 24 points, which has been used as a cutoff value for cognitive function (Folstein et al., 1975), and the TUG scores were below 13.5 s, which has been used as a cutoff value for motor function (Shumway-Cook et al., 2000).

**Table 1:** Participants' details.

	**Older adults**			**Younger adults**		
	**Intervention group (*n* = 10)**	**Control group (*n* = 11)**	***p-*value**	**Intervention group (*n* = 10)**	**Control group (*n* = 9)**	***p-*value**
Gender (male/female)^a^	4/6	5/6	n.s.	8/2	8/1	n.s.
Age (years)^b^	74.5 ± 5.4	74.4 ± 5.5	n.s.	23.0 ± 3.3	27.4 ± 5.8	n.s.
Height (cm)^b^	159.2 ± 9.8	163.9 ± 10.6	n.s.	168.3 ± 7.9	171.2 ± 6.2	n.s.
Weight (kg)^b^	56.5 ± 12.4	58.8 ± 13.4	n.s.	61.3 ± 8.0	60.9 ± 12.4	n.s.
Shoulder width (cm)^b^	37.85 ± 2.89	38..05 ± 4.50	n.s.	40.4 ± 2.8	41.3 ± 1.2	n.s.
MMSE (points)^b^	29.7 ± 0.48	29.0 ± 2.05	n.s.	–	–	–
TUG (s)^b^	6.89 ± 0.96	6.11 ± 1.03	n.s.	–	–	–

*MMSE, Mini–Mental State Examination; TUG, Timed Up and Go test*.

a *Pearson's chi-squared test*.

b *Mann–Whitney U-test*.

### Collision Avoidance Behavior

**Figure 5:**
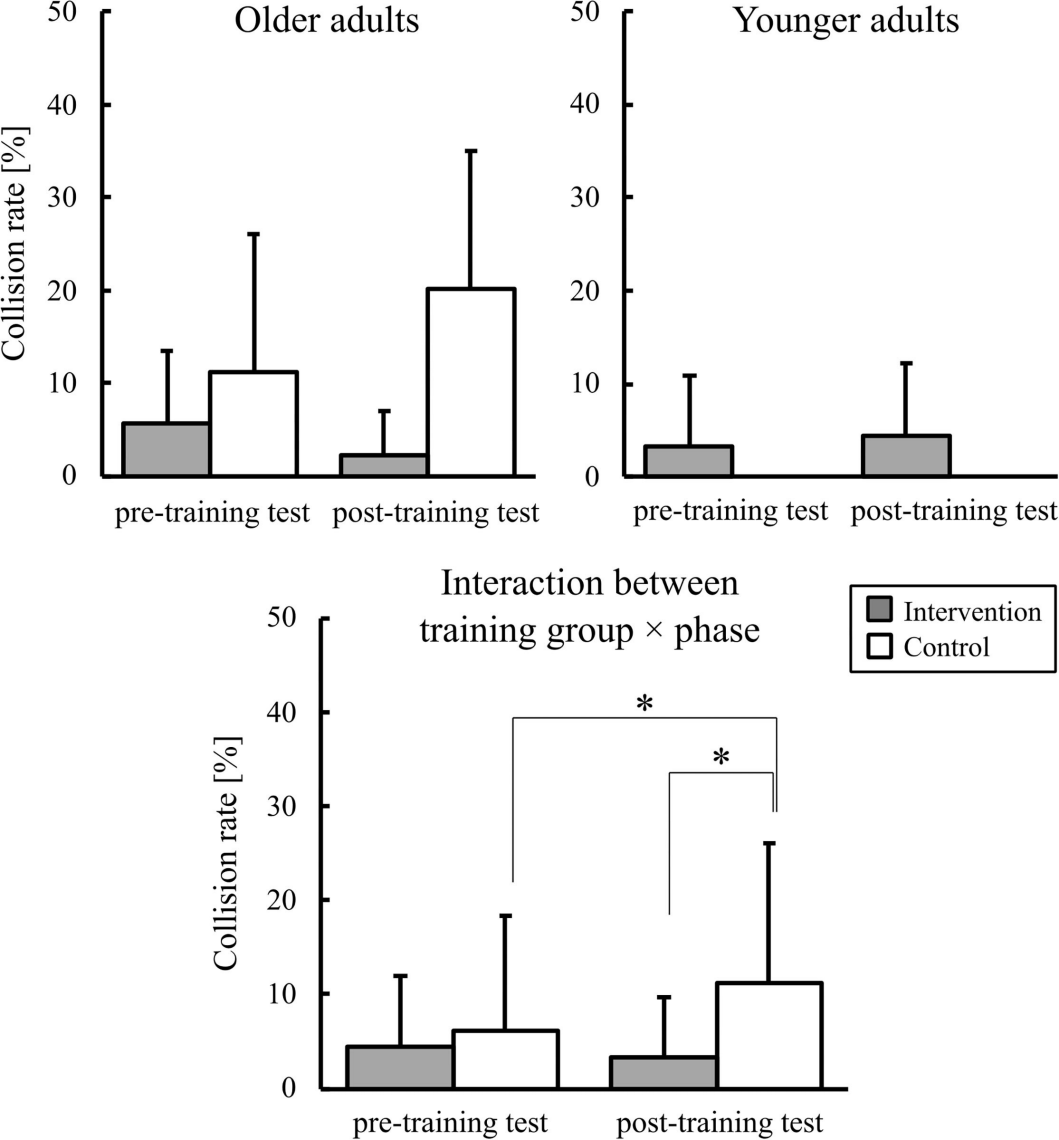
Graphs of the collision rate. Error bars represent the standard deviation among participants. * indicates the significant difference.

The mean collision rate is shown in Figure 5. Because we used an arcsine-transformed data for the statistical analysis, we present an additional figure as Supplementary Figure 1 to show that the distribution of the transformed data was comparable to that of the original one. An ANOVA for the adjusted data using the arcsine transformation showed a significant two-way interactions of the training group × phase [*F*_(1,36)_ = 7.37, *p* < 0.01, $\eta_{p}^{2}$ = 0.17]. To test the study's hypothesis, *post-hoc* comparisons of the interactions were performed. During the post-training test, the mean collision rate in the control group was significantly higher than that in the intervention group. In the control group, the mean collision rate in the pre-training test was significantly higher than that in the post-training test. Two-way interactions of age × training group [*F*_(1,36)_ = 7.97, *p* < 0.01, $\eta_{p}^{2}$ = 0.18], three-way age × training group × phase interaction [*F*_(1,36)_ = 12.24, *p* < 0.01, $\eta_{p}^{2}$ = 0.25], and a main effect of age [*F*_(1,36)_ = 8.95, *p* < 0.01, $\eta_{p}^{2}$ = 0.19] were also significant.

**Figure 6:**
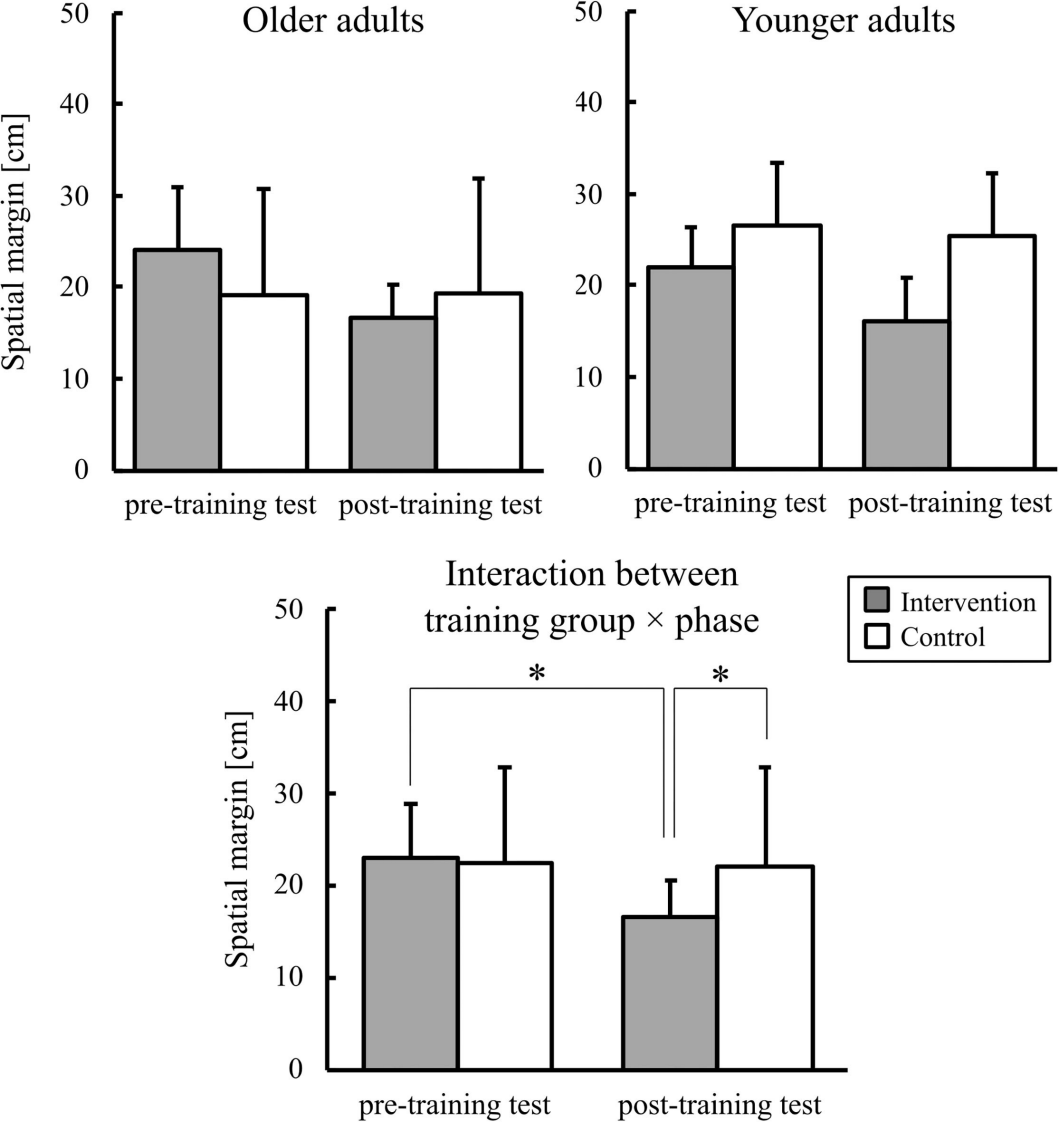
Graphs of the mean spatial margin. Error bars represent the standard deviation among participants. * indicates the significant difference.

The mean spatial margin when crossing the aperture is shown in Figure 6. An ANOVA showed a significant two-way training group × phase interaction [*F*_(1,36)_ = 15.02, *p* < 0.01, $\eta_{p}^{2}$ = 0.29]. *Post-hoc* comparisons showed that, in the intervention group, the mean spatial margin was significantly smaller in the post-training test than in the pre-training test. During the post-training test, the spatial margin was significantly smaller in the intervention group than that in the control group. A significant main effect of phase was also found [*F*_(1,36)_ = 19.65, *p* < 0.01, $\eta_{p}^{2}$ = 0.35].

**Figure 7:**
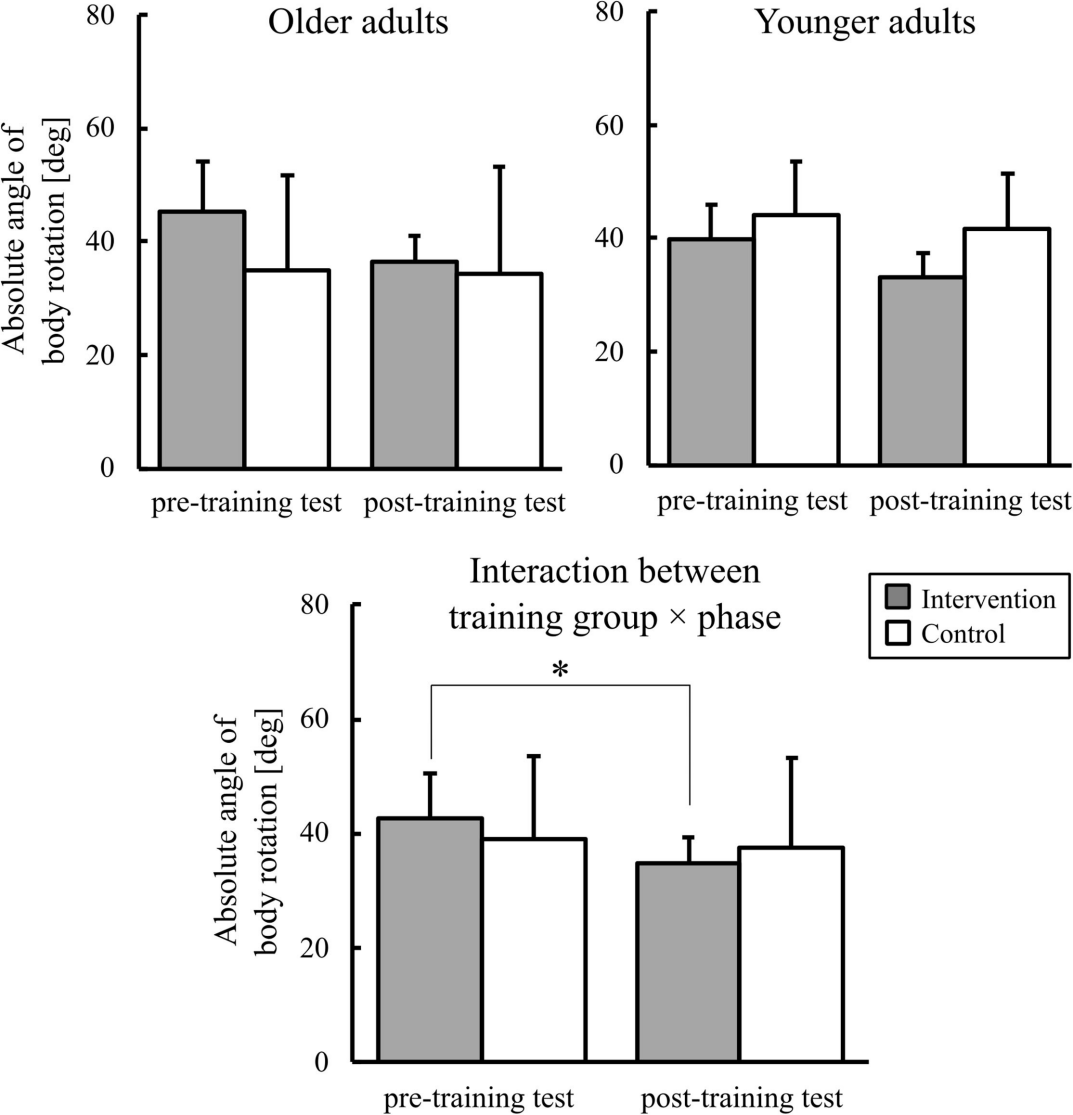
Graphs of the mean absolute angle of body rotation. Error bars represent the standard deviation among participants. * indicates the significant difference.

The mean absolute angle of body rotation is shown in Figure 7. An ANOVA showed a significant two-way training group × phase interaction [*F*_(1,36)_ = 10.55, *p* < 0.01, $\eta_{p}^{2}$ = 0.23]. *Post-hoc* comparisons showed that, in the intervention group, the mean body-rotation angle was significantly smaller in the post-training test than in the pre-training test. A significant main effect of phase was also found [*F*_(1,36)_ = 25.44, *p* < 0.01, $\eta_{p}^{2}$ = 0.41].

**Figure 8:**
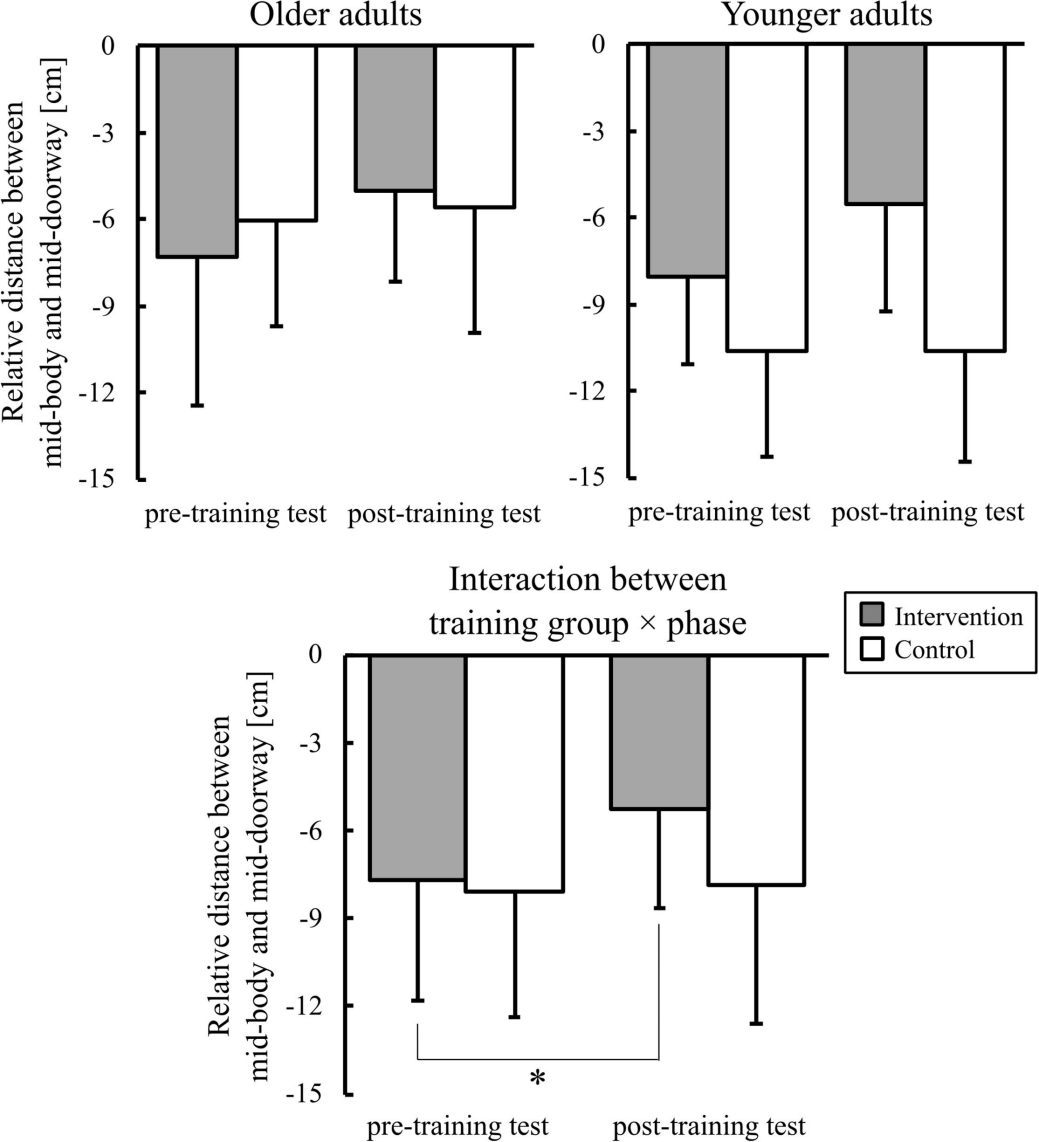
Graphs of the relative distance between mid-body and mid-doorway. Error bars represent the standard deviation among participants. * indicates the significant difference.

The relative distance between mid-body and mid-doorway is shown in Figure 8. An ANOVA showed a significant two-way training group × phase interaction [*F*_(1,36)_ = 6.89, *p* < 0.05, $\eta_{p}^{2}$ = 0.16]. *Post-hoc* comparisons showed that, in the intervention group, the relative distance was significantly greater in the post-training test than in the pre-training test. During the post-training test, the relative distance was significantly greater in the intervention group than that in the control group. There were also significant main effects of age [*F*_(1,36)_ = 9.35, *p* < 0.01, $\eta_{p}^{2}$ = 0.21] and phase [*F*_(1,36)_ = 5.63, *p* < 0.05, $\eta_{p}^{2}$ = 0.14].

### Quality of VR Training of Participants in the Intervention Group

**Figure 9:**
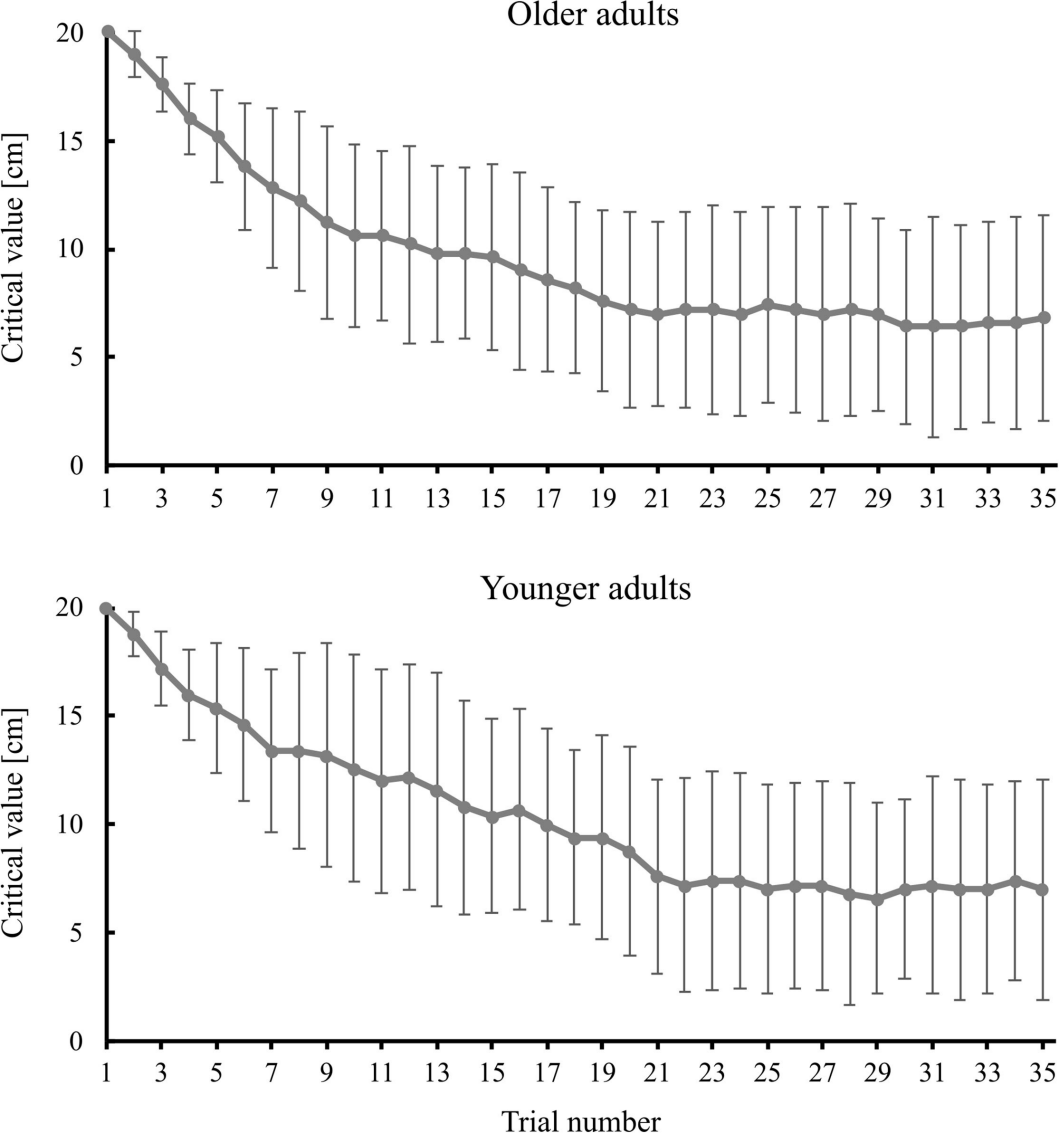
The mean value of the critical value for older and younger adults. Error bars represent the standard deviation among participants.

In the older adults, the mean of critical values for each practice trial in the intervention group is shown in Figure 9. Visual inspection of the figure shows that the critical values gradually became smaller until around trail 20. The number of positive feedback signals in a total of 35 practice trials was 11.1 ± 3.1. Of these, 7.8 ± 1.8 occurred in the first half (trials 1–17), and 3.3 ± 1.9 occurred in the second half (trials 18–35). The total number of unsuccessful trials (i.e., trials in which collisions occurred) observed for older participants was 38. In 36 of 38 trials (94.7%), the collision occurred at the “trailing” side of the body (i.e., the side of the body opposite that with which they approached an aperture with body rotation). The mean angle of body rotation was 33.84 ± 14.94 deg., which was slightly smaller than that for successful trials (38.14 ± 16.10 deg.). The relative distance between mid-body and mid-doorway was −9.21 ± 5.69 cm, which was slightly smaller than that for successful trials (−6.02 ± 4.96 cm).

In the younger adults, the mean of critical values for each practice in the intervention group is shown in Figure 9. The trend was similar to that in older adults. The number of positive feedback signals from a total of 35 practice trials was 11.9 ± 3.1. Of these, 7.6 ± 2.2 occurred in the first half (trials 1–17), and 4.3 ± 1.8 occurred in the second half (trials 18–35). The total number of unsuccessful trials observed for the younger participants was seven. All of these occurred on the trailing side of the body. The mean angle of body rotation was 45.01 ± 5.41 deg., which was slightly greater than that for successful trials (39.64 ± 10.86 deg.). The relative distance between mid-body and mid-doorway was −13.68 ± 2.14 cm, which was slightly smaller than that for successful trials (−8.61 ± 4.84 cm).

## Discussion

The results showed that, for both older and younger participants in the intervention group, the improvement in collision-avoidance behavior observed during training in the VR environment was transferred to collision-avoidance behavior in the real environment. During training, the mean critical value of the spatial margin gradually decreased (from 20 cm to <10 cm on average) for both older and younger participants (Figure 9). These results showed that, regardless of age, participants in the intervention group improved their skill to pass through the aperture while minimizing the spatial margin and maintaining a state of safety. This suggests that they improved their ability to safely and efficiently avoid collision. Comparisons of the collision-avoidance behavior between pre- and post-training test performance showed that the spatial margin became significantly smaller, while the collision rate remained unchanged. This suggests that the improvement in behavior obtained under the VR environment was successfully transferred to the real environment. Such a tendency was not observed in the control group who had experimented stepping in place without performing the collision avoidance behavior during training. As a result, for both older and younger adults, neither the spatial margin nor the collision rate improved. These results lead us to conclude that, regardless of age, the protocol of the VR training used in the present study was effective to transfer the behavior learned in a VR environment to a real environment.

The present findings were consistent with relevant studies demonstrating the effects of VR training. Previous studies have shown that adding vibratory stimulation might improve the realism of the VR environment (Grassini et al., 2021) and to enhance the motor-learning effect (Grassini et al., 2020). Positive feedback for successful trials effectively reinforces participants' behavior (Abe et al., 2011; Nikooyan and Ahmed, 2015; Vassiliadis et al., 2021). One reason for this effect is speculated to be that positive feedback enhances motivation, leading to an enhanced motor-learning effect (Wulf and Lewthwaite, 2016). A possible future study to validate this speculation would compare skill transfer between the intervention group in the present study and the control group in which positive feedback could be given for all trials regardless of the participant's behavior. The gradual increase in task difficulty during training not only reduces cognitive demand (Sawers and Hahn, 2013; Sawers et al., 2013) but also provides task difficulty according to the skill level of the individual performing the task (Kagerer et al., 1997; Guadagnoli and Lee, 2004; Akizuki and Ohashi, 2015). Considering these findings, we believe that modifications of the VR training to enrich feedback and gradually increase task difficulty could enhance the training effect.

Detailed analyses of collision avoidance behavior showed similarities and dissimilarities between older and younger adults. The pattern of changes in the mean critical value during training, which indicates the improvement in behavior during training, was similar (Figure 9): the mean critical value gradually decreased until the trial 20; it improved from 20 cm to <10 cm on average; and positive feedback was provided more frequently in the first 17 trials. Improvement of the collision avoidance behavior in the post-test was also similar; smaller spatial margin, smaller magnitude of body rotation angle greater distance between mid-body and mid-doorway (i.e., mid-body was deviated away from the approaching side) was observed (Figures 6–8).

In contrast, the behavioral characteristics of unsuccessful trials during VR training were slightly different between the older and younger adults. First, the total number of collisions experienced was different (38 vs. 7 trials for older and younger participants, respectively). For older participants, collisions occurred with smaller angles of body rotation and shifting the mid-body farther from the side at which participants entered into the aperture. This resulted in collisions on the other side, that is, the side of the body facing backward during the body rotation. It seemed that modifying body-rotation behavior through training was easier for older participants than adjusting the location of the back side of the body—the “invisible” side—to avoid collision. In contrast, for younger participants, relatively greater body rotation angle was observed for unsuccessful trials. This may suggest that other factors, such as delayed timing of rotating the body may have improved through training. These findings suggest that, although both older and younger adults showed similar improvement of their collision-avoidance behavior throughout the VR training, the results of their collision experiences during training were different between the age groups.

The originality of the VR training used in the present study, as well as in Kondo et al. (2021), was to request that participants perform the collision-avoidance behavior not only safely but also efficiently. There are a number of studies demonstrating that older adults have a tendency to adopt a cautious strategy, such as creating a greater safety margin when avoiding obstacles (Lu et al., 2006; Gérin-Lajoie et al., 2008; Hackney and Cinelli, 2011, 2013; Muir et al., 2015, 2019). A cautious strategy may be useful in preventing collision; however, it may disturb the maintenance of balance at the moment of obstacle avoidance (Muir et al., 2015; Yamagata et al., 2021). Moreover, a cautious strategy may prevent older adults from fine-tuning their behavior in response to environmental changes (Blakemore et al., 2002). Because a cautious strategy allows older adults to avoid any characteristic of obstacles with the single, exaggerated movement pattern (i.e., a “one size fits all” pattern), older adults showing the cautious strategy may be free from performing fine-tune of their behavior in response to environmental constraints in their daily locomotor activities. We believe this will eventually be detrimental to their capacity for adaptive locomotor adjustment. Sakurai and colleagues showed that, in older adults, the perception of one's own action boundary became less accurate (overestimation of their own ability to step over an obstacle) as the frequency of going outdoors became lower (Sakurai et al., 2021). This suggests that the experience of fine-tuning behavior in response to environmental changes is important to maintain the perceptual ability, and possibly also the motor ability, to adjust locomotor patterns. The protocol of asking participants to avoid collisions with minimum spatial margin can lead them to alter their behavior in response to the obstacle characteristics (e.g., the width of an aperture or the height of a doorstep). We therefore believe that the protocol would help to induce fine-tuning of their behavior.

There were several limitations of this study. First, the effects of the three modifications that were newly introduced to enhance the training effect (the addition of vibratory stimulation, positive feedback for successful trials, and a gradual increase in task difficulty during training) were not examined. With this limitation, we are unable to discuss whether each of the modifications had a significant impact on the training effect. Second, the learning effect was investigated only immediately after the VR training. Some studies showed that retention effects were more beneficial for motor learning (Kantak and Winstein, 2012; Gray, 2018). Future studies should examine whether the transfer could be sustained longer. Third, although we showed skill transfer from the VR environment to the real environment, this does not ensure that the behavior learned through lab-based training is generalizable to real-life behavior. Future studies need to test whether improvement of collision-avoidance behavior in both safety and efficiency is transferred to basic locomotor activities. Fourth, the sample size was small. More than 46 sample size would be necessary to validate the study's conclusions (calculated based on the power analyses with G^*^Power: effect size = 0.06, significant threshold = 0.05 and power levels = 0.9). Finally, because the intervention and control groups were not matched on the basis of their pre-training tests, the equality of the groups was not ensured. Although no significant differences were observed in the pre-test between the groups, this may have been due to the small sample size.

In conclusion, our results showed that, regardless of age, the VR training used in the present study contributed to improving collision-avoidance behavior in older adults. This suggests that the behavior improvement obtained while stepping in place, instead of walking, in a VR environment was transferable to the behavior while walking in a real environment. The physical demand of stepping in place would be lower than that of walking. Moreover, in our VR system, participants could observe the VR image while standing still and perform the collision-avoidance behavior at the time of crossing. This would be advantageous in applying the system to those who have difficulty walking. This would also be helpful in planning a training program in which a relatively large number of collision-avoidance experiences are necessary, considering that the number of trials to train while walking would be limited due to the effects of fatigue.

## Data Availability Statement

The original contributions presented in the study are included in the article/Supplementary Material, further inquiries can be directed to the corresponding author/s.

## Ethics Statement

The studies involving human participants were reviewed and approved by the Ethics Committee of Tokyo Metropolitan University. The patients/participants provided their written informed consent to participate in this study.

## Author Contributions

YS: conceptualization, methodology, data curation, formal analysis, and writing–original draft. KF: software and methodology. KS: data curation and formal analysis. TH: conceptualization, methodology, and writing–review and editing. All authors contributed to the article and approved the submitted version.

## Funding

TH and KF were funded by the Japan Society for the Promotion of Science (16H06325 and 18K10890, respectively).

## Conflict of Interest

The authors declare that the research was conducted in the absence of any commercial or financial relationships that could be construed as a potential conflict of interest.

## Publisher's Note

All claims expressed in this article are solely those of the authors and do not necessarily represent those of their affiliated organizations, or those of the publisher, the editors and the reviewers. Any product that may be evaluated in this article, or claim that may be made by its manufacturer, is not guaranteed or endorsed by the publisher.
