# Supplementary figures and images for: Improved Walking Through an Aperture in a Virtual Environment Transfers to a Real Environment: Introduction of Enriched Feedback and Gradual Increase in Task Difficulty
Source: Front Sports Act Living. 2022 Mar 4;4:844436. doi: 10.3389/fspor.2022.844436 (PMC8931267; doi:10.3389/fspor.2022.844436)

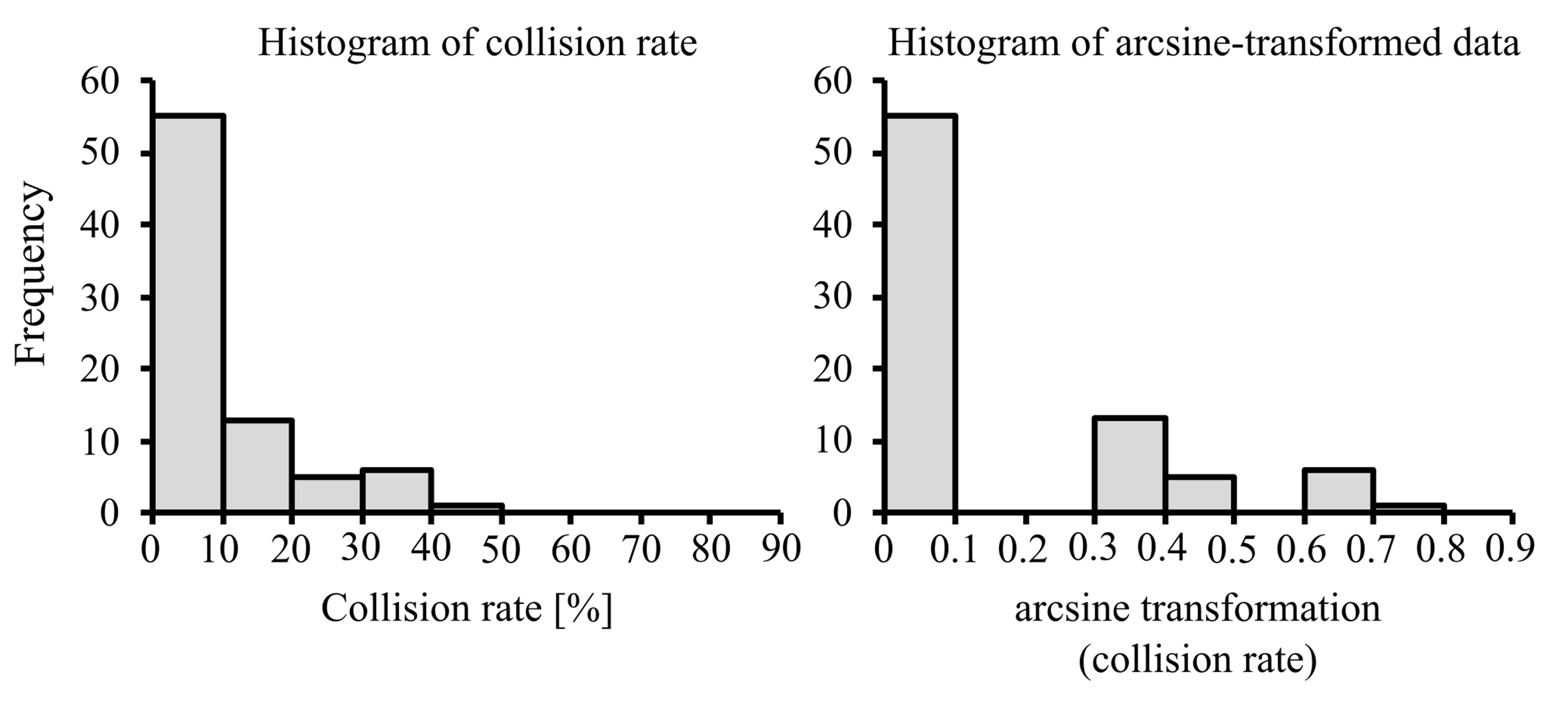

Supplement: Supplementary file 1 [file Image_1.TIFF]
